# Bt in the Spotlight: Defending Its Relevance in an RNAi-Driven Future

**DOI:** 10.3390/insects16080837

**Published:** 2025-08-14

**Authors:** Camilo Ayra-Pardo, Denis J. Wright

**Affiliations:** 1 CIIMAR/CIMAR LA, Interdisciplinary Centre of Marine and Environmental Research, University of Porto, Terminal de Cruzeiros do Porto de Leixões, Avda. General Norton de Matos s/n, 4450208 Matosinhos, Portugal; 2 Department of Life Sciences, Faculty of Natural Sciences, Imperial College London, Silwood Park Campus, Ascot, Berkshire SL5 7PY, UK

**Keywords:** *Bacillus thuringiensis*, RNA interference, bioinsecticides, sustainable agriculture, integrated pest management

## Abstract

The rise of RNAi-based biopesticides marks a turning point in pest control, prompting reflection on the future role of *Bacillus thuringiensis* (Bt). Far from being outdated, Bt remains a cornerstone of sustainable agriculture, with proven efficacy, safety, and public trust. This opinion piece advocates for Bt’s continued relevance in an RNAi-driven future, urging a complementary, not competitive, view of both technologies within integrated pest management frameworks.

## Simple Summary

While new pest control methods using RNAi technology provide more precise ways to fight harmful insects, the long-used entomopathogenic bacterium *Bacillus thuringiensis* (Bt) remains an essential component of sustainable pest management due to its safety, effectiveness, and wide acceptance. This opinion piece suggests that Bt and RNAi should be used in combination, not in competition, to improve how we protect crops sustainably.

## 1. Why Bt Endures

For decades, the biopesticide *Bacillus thuringiensis* (Bt) has been a cornerstone of sustainable pest management [1], primarily through its production of crystal (Cry) and vegetative insecticidal proteins (Vip) that target specific insect groups while exerting minimal impact on non-target organisms and the environment [2,3]. Originally applied as spore crystal formulation sprays containing Cry toxins and later through the genetic engineering of crops to express Cry and Vip toxins [4], Bt has significantly reduced reliance on conventional synthetic insecticides while providing extended control of target species via constitutive toxin expression in engineered crops [5]. Moreover, the use of pro-active resistance management strategies such as gene pyramiding and spatial refuges has helped to delay the evolution of resistance to Bt crops [6]. Bt’s safety, affordability, and broad applicability have contributed to its widespread adoption in both conventional and organic farming systems [2,7]. Its enduring success stems from a combination of field efficacy, formulation stability, and a vast body of environmental risk assessments underpinning regulatory approval worldwide [8,9]. Despite all this, challenges such as resistance evolution and emergence of secondary pests must be acknowledged. For example, resistance to Bt Cry proteins has been documented in pests like pink bollworm (*Pectinophora gossypiella*) and fall armyworm (*Spodoptera frugiperda*), and secondary pest outbreaks, such as mirid bugs (Heteroptera: Miridae), which became major pests in cotton and adjacent crops due to reduced chemical control [10,11]. This Special Issue, “*Recent Advances in Bt Insecticides and Bt Crops: Challenges and Opportunities*”, seeks to compile cutting-edge research on novel Bt strains, toxin mechanisms, resistance management, and ecological impacts, providing a comprehensive overview of the current state and prospects of Bt technology in sustainable agriculture.

## 2. RNAi’s Rise

The December 2023 commercial approval of the sprayable RNA interference (RNAi)-based insecticide Ledprona (marketed as Calantha^TM^) by the U.S. Environmental Protection Agency (EPA) under Section 3 of the Federal Insecticide, Fungicide, and Rodenticide Act (FIFRA) [12] marked a pivotal shift in pest control. Ledprona targets the Colorado potato beetle (*Leptinotarsa decemlineata*)—a major pest with well-documented resistance to both conventional insecticides and to Bt toxins [13,14]—disrupting a landscape long dominated by Bt-based bioinsecticides. Supported by over 200 publicly available field trials conducted across U.S. potato-growing regions, Ledprona demonstrated consistent efficacy at field application rates as low as ~4 g/acre (~9 g/ha), with negligible non-target impacts even at doses 100-fold higher than the recommended field rate [15].

**Figure 1:**
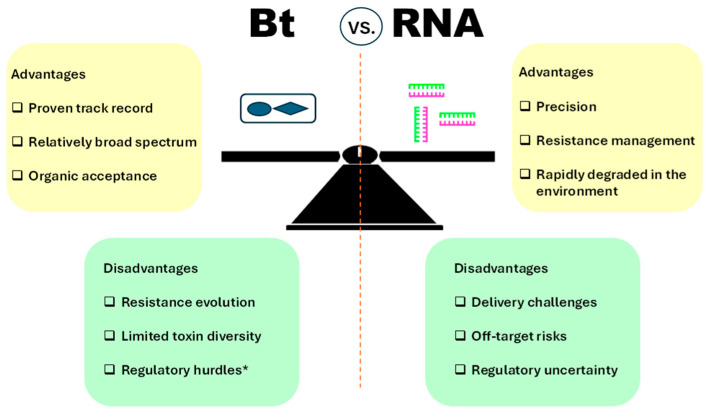
Comparative overview of Bt and RNA-based biopesticides. The diagram contrasts the main advantages and disadvantages of *Bacillus thuringiensis* (Bt)-based and RNA interference (RNAi)-based pest control strategies. The central balance illustration emphasises the complementary potential and trade-offs of both technologies in integrated pest management. * Genetically modified (GM) crops including Bt crops are banned in some countries, particularly within Europe.

RNAi stands out for its precision, adaptability across pest species and delivery platforms, and rapid design. The RNAi mechanism introduces a revolutionary approach to insect control by enabling sequence-specific suppression of essential gene expression [16]. Once an essential gene is identified in a target pest, synthetic double-stranded RNA (dsRNA) can be designed and produced with high fidelity. This modularity—referring to the ease of redesigning dsRNA sequences while retaining the same delivery framework—allows RNAi biopesticides to keep pace with evolving pest pressures, making them ideal for addressing newly invasive or resistant species. The RNAi approach has also been extended to transgenic crops for commercial purposes [17], where RNAi traits are expressed directly in the plant for season-long protection. To determine whether RNAi can outperform Bt in integrated pest management (IPM), their respective strengths and limitations must be evaluated. Comparative advantages and trade-offs of RNAi relative to Bt are summarized in Figure 1.

RNAi technologies face technical and regulatory challenges, including delivery efficiency, environmental stability, off-target effects, and high production costs, some of which Bt has addressed over decades of use. Also, RNAi efficacy is highly variable across insect orders, with some species, particularly Lepidoptera, showing limited response due to inefficient dsRNA uptake or rapid degradation [18,19]. Formulating RNAi products that resist degradation in the environment and in the insect gut and achieve effective delivery inside insect cells remains an ongoing challenge. While advances such as nanoparticle encapsulation [20], liposomes [21], and biopolymer matrices [22] are promising, these add cost and complexity. In contrast, Bt already benefits from highly stable fermentation products that are easy to apply and remain effective for extended periods under standard agricultural conditions [7]. Recent progress by GreenLight Biosciences, which has developed a cell-free biomanufacturing platform that significantly lowers dsRNA production costs while increasing yield and scalability represents an important step toward overcoming economic barriers to commercial RNAi applications in pest control [23].

Another critical consideration is the development of resistance. While gene pyramiding and refuge strategies have helped delay Bt resistance in some cases [24,25], recent studies show that insects like the Colorado potato beetle can also develop resistance to RNAi with minimal fitness cost [26]. Though the exact resistance mechanisms remain uncharacterized, RNAi resistance in other chrysomelids, the western corn rootworm (*Diabrotica virgifera virgifera* LeConte), was linked to reduced uptake of dsRNA in the midgut cells [27]. Interestingly, no resistance to a Bt Cry3Bb1 protein was observed, confirming RNAi and Bt Cry proteins operate via completely different mechanisms. This emerging risk demands proactive resistance management strategies for RNAi akin to those already in place for Bt.

## 3. Converging Paths

A promising development in pest control is the convergence of Bt and RNAi technologies within transgenic platforms. By pyramiding RNAi traits with Bt genes, engineered plants can simultaneously target Bt-resistant pests, enabling broader and more durable protection [28]. For example, SmartStax^®^ PRO maize—commercially deployed since 2022—combines Bt proteins Cry3Bb1 and Cry34Ab1/Cry35Ab1 (now reclassified as Gpp34/Tpp35Ab1 [29]) with a dsRNA targeting the *dvSnf7* gene (essential for cellular vesicle transport), demonstrating field efficacy against western corn rootworm, including Bt-resistant populations [30]. Similarly, experimental cotton pyramids co-expressing a Cry1Ac/Cry1Ab chimeric protein with RNAi targeting juvenile hormone synthesis genes (*JHAMT* or *JHBP*) reduced survival of Bt-resistant *Helicoverpa armigera* and delayed resistance evolution in simulations, relative to Bt-only cotton [31]. This combined strategy may help preserve the effectiveness of stacked traits over time and exemplifies how combining independent modes of action—Bt’s gut disruption and RNAi’s essential gene silencing—can reinforce IPM frameworks. However, combining Bt and RNAi traits has raised concerns about regulatory complexity, extended approval timelines, and increased development costs, which may delay real-world deployment [32]. Moreover, public perception varies widely between regions and technologies.

## 4. Regulatory Hurdles

The regulatory landscape for RNAi products remains fragmented, exemplified by the stark contrast between the EU’s precautionary GMO classification versus the USA EPA’s case-by-case approach for non-transgenic RNAi. In the EU, regulatory agencies apply particularly strict environmental risk assessments to RNAi technologies, due in part to uncertainties surrounding their environmental persistence and potential for off-target gene silencing [33]. This reactive framework mirrors Bt’s early regulatory challenges but faces higher stakes given RNAi’s rapid technological evolution [34]. Critics contend RNAi-based pesticides should be regulated like genetically modified organisms (GMOs), citing potential heritable gene silencing effects and ecological risks not fully addressed by current frameworks [35]. RNAi’s modular design requires agile, anticipatory governance to balance innovation with ecological risk management, particularly as novel formulations (e.g., nanoparticle-encapsulated dsRNA) challenge traditional risk assessment paradigms [36]. Unlike transgenic Bt crops, topical RNAi sprays are non-GM and biodegradable—advantages that could enhance public acceptance. However, recent surveys reveal persistent consumer confusion, with many conflating RNAi with genetic modification, particularly in regions where GMOs remain controversial [37,38,39]. Older demographics (aged >50) and organic producers show cautious interest, but trust hinges on transparent risk communication and clear labeling, areas where current regulatory frameworks are inadequate. Without global coordination, divergent regulatory standards will perpetuate market asymmetries and erode hard-won public trust in biopesticides [40].

## 5. Future Directions

Bt has withstood the test of time, adapting through the use of a wider range of protein classes, improved formulations, and incorporation into transgenic crops, while Bt sprays remain an important tool in IPM and organic agriculture. However, Bt technology also faces limitations, including resistance evolution in target pests, the emergence of secondary pests, variability in regulatory approval across regions, and uneven adoption in some geographies. The arrival of RNAi technologies is a complementary advancement to Bt—one that brings gene-level precision, adaptability to resistance, and the opportunity for tailored pest solutions. The future of pest control lies not in a binary choice between Bt and RNAi, but in their strategic integration to build resilient, sustainable IPM systems. As this Special Issue seeks to demonstrate, the Bt research community continues to innovate and evolve, and its contributions remain central to shaping the future of biological pest control.

## Data Availability

No new data were created or analyzed in this study. Data sharing is not applicable to this article.

## Abbreviations

The following abbreviations are used in this manuscript:

Bt	*Bacillus thuringiensis*
Cry	Crystal
Vip	Vegetative insecticidal protein
RNAi	RNA interference
dsRNA	Double-stranded RNA
GM	Genetically modified
EU	The European Union
USA	The United States of America
EPA	Environmental Protection Agency
GMOs	Genetically modified organisms
